# Age-Related Variation in Sympathetic Nerve Distribution in the Human Spleen

**DOI:** 10.3389/fnins.2021.726825

**Published:** 2021-10-14

**Authors:** Cindy G. J. Cleypool, David J. Brinkman, Claire Mackaaij, Peter G. J. Nikkels, Martijn A. Nolte, Misha D. Luyer, Wouter J. de Jonge, Ronald L. A. W. Bleys

**Affiliations:** ^1^Division of Surgical Specialties, Department of Anatomy, University Medical Center Utrecht, Utrecht University, Utrecht, Netherlands; ^2^Tytgat Institute for Liver and Intestinal Research, Amsterdam University Medical Center, University of Amsterdam, Amsterdam, Netherlands; ^3^Department of Surgery, Catharina Hospital, Eindhoven, Netherlands; ^4^Division of Laboratories, Pharmacy, Biomedical Genetics and Pathology, Department of Pathology, University Medical Center Utrecht, Utrecht, Netherlands; ^5^Department of Molecular and Cellular Hemostasis, Sanquin Research and Landsteiner Laboratory, Amsterdam, Netherlands; ^6^Department of Surgery, University Hospital Bonn, Bonn, Germany

**Keywords:** spleen, sympathetic innervation, neuroimmunomodulation, periarteriolar lymphatic sheath, cholinergic anti-inflammatory pathway

## Abstract

**Introduction:** The cholinergic anti-inflammatory pathway (CAIP) has been proposed as an efferent neural pathway dampening the systemic inflammatory response via the spleen. The CAIP activates the splenic neural plexus and a subsequent series of intrasplenic events, which at least require a close association between sympathetic nerves and T cells. Knowledge on this pathway has mostly been derived from rodent studies and only scarce information is available on the innervation of the human spleen. This study aimed to investigate the sympathetic innervation of different structures of the human spleen, the topographical association of nerves with T cells and age-related variations in nerve distribution.

**Materials and Methods:** Spleen samples were retrieved from a diagnostic archive and were allocated to three age groups; neonates, 10–25 and 25–70 years of age. Sympathetic nerves and T cells were identified by immunohistochemistry for tyrosine hydroxylase (TH) and the membrane marker CD3, respectively. The overall presence of sympathetic nerves and T cells was semi-automatically quantified and expressed as total area percentage. A predefined scoring system was used to analyze the distribution of nerves within different splenic structures.

**Results:** Sympathetic nerves were observed in all spleens and their number appeared to slightly increase from birth to adulthood and to decrease afterward. Irrespective to age, more than halve of the periarteriolar lymphatic sheaths (PALSs) contained sympathetic nerves in close association with T cells. Furthermore, discrete sympathetic nerves were observed in the capsule, trabeculae and red pulp and comparable to the total amount of sympathetic nerves, showed a tendency to decrease with age. No correlation was found between the number of T cells and sympathetic nerves.

**Conclusion:** The presence of discrete sympathetic nerves in the splenic parenchyma, capsule and trabecular of human spleens could suggest a role in functions other than vasoregulation. In the PALS, sympathetic nerves were observed to be in proximity to T cells and is suggestive for the existence of the CAIP in humans. Since sympathetic nerve distribution shows interspecies and age-related variation, and our general understanding of the relative and spatial contribution of splenic innervation in immune regulation is incomplete, it remains difficult to estimate the anti-inflammatory potential of targeting splenic nerves in patients.

## Introduction

The cholinergic anti-inflammatory pathway (CAIP) comprises an efferent neural pathway that dampens the systemic inflammatory response via the spleen and is suggested to involve sequential activation of the efferent vagus nerve and the splenic plexus (Reardon, 2016; Pavlov and Tracey, 2017). Others have put forward that instead of the efferent vagus nerve, this pathway involves the greater splanchnic nerve (Komega et al., 2018). Irrespective, activation of the splenic plexus results in a cascade of intrasplenic events, starting with the release of norepinephrine (NE) (Kees et al., 2003). Studies have demonstrated that NE then activates adrenergic receptors on CD4^+^ ChAT^+^ T lymphocytes (Rosas-Ballina et al., 2011; Vida et al., 2011, 2017), which in turn produce and secrete acetylcholine (ACh) (Borovikova et al., 2000; Rosas-Ballina et al., 2011). ACh then inhibits the release of the pro-inflammatory cytokine tumor necrosis factor alpha (TNFα) from activated macrophages via nicotinergic receptor signaling (Borovikova et al., 2000; de Jonge et al., 2005; Kox et al., 2009; Lu and Kwan, 2014).

Morphological evidence for the presence of sympathetic nerves in proximity to splenic T lymphocytes was provided earlier by Bellinger et al. (1987, 1992). In a study on rat spleens, they observed sympathetic nerves diverging into T lymphocyte specific white pulp areas, also known as periarteriolar lymphatic sheaths (PALSs). In the PALSs these nerves were in close proximity to T lymphocytes and formed synaptic connections (Felten and Olschowka, 1987). The presence of sympathetic nerves which could release NE in the proximity of T lymphocytes in the human spleen might hold potential as a therapeutic target for immune related disease and knowledge on the anatomical configuration of splenic innervation in humans is therefore essential.

The presence of sympathetic nerves at the medio adventitial junction in human spleens has been described in various studies (Heusermann and Stutte, 1977; Kudoh et al., 1979; Anagnostou et al., 2007; Verlinden et al., 2018), however, innervation of T cell specific lymphoid tissue has only been reported once (Hoover et al., 2017). In the latter study, sympathetic innervation patterns in spleens of end-stage sepsis patients were investigated and sympathetic nerves were observed to be in close association with lymphocytes in the PALS of the control group (trauma patients who died after hemorrhagic stroke). The results of this study were descriptive and it remains unclear whether this was a common feature and observed in all PALSs, or only occasionally. Since, PALS related sympathetic nerves were seldom observed in end-stage sepsis patients, the authors suggested this difference to be disease-related (Hoover et al., 2017). However, other factors, such as aging, are known to contribute to decline of sympathetic innervation as well, as shown in the rat spleen (Bellinger et al., 1987, 1992) and human cerebral arteries (Bleys and Cowen, 2001). If human splenic innervation is subject to age-related decline as well, this information is of relevance because it might determine the window of application of anti-inflammatory neuromodulation along age. Since the age profile of a substantial number of patients of the control group in the study of Hoover et al. (2017) was lacking, as well as comprehensive data on the prevalence of PALS related sympathetic nerves, our understanding of human splenic innervation remains incomplete.

Therefore, in this study, quantitative and semi-quantitative analytical methods were used to investigate the distribution of sympathetic nerves in human spleens of various age groups. Although the PALS is considered to represent the primary structure of T cell neuromodulation, T cells migrate through the spleen and exposure to NE might occur at any location they pass while entering or exiting the spleen. Therefore, blood vessels, red pulp, trabeculae and the capsule were evaluated for the presence of sympathetic nerve tissue as well.

## Materials and Methods

### Tissue Samples

A total of 26 paraffin embedded splenic samples were provided the Pathology Department of the University Medical Center Utrecht. Samples were divided into three age groups, being 40 weeks of gestation (from now on referred to as neonatal), 10–25 years and 25–70 years. This study was approved by the Medical Ethical Committee (#18-167) as a “non-Medical Research Act” study and the Biobank of the University Medical Center Utrecht approved to use the rest biomaterial for this research (biobank #18-284). None of the individuals was known with immunological or splenic clinical conditions. Table 1 contains data on age, sex, and cause of death.

**TABLE 1:** Patient profiles.

**#**	**Sex**	**Age**	**Cause of death**
**25–70 years (*N* = 7)**			
1	F	46	Pancreatic tail cyst
2	F	66	Myocardial infarct
3	F	52	Subarachnoid hemorrhage
4	F	48	Traumatic motor bike accident
5	M	66	Unknown
6	M	26	Arrhythmia
7	M	29	Long QT syndrome
**10–25 years (*N* = 7)**			
8	F	14	Acute unexpected death, probably due to cardiac arrest
9	M	16	Arrhythmia
10	M	11	Sudden unexpected death due to coronary artery anomaly
11	M	11	New diabetes mellitus with keto-acidosis
12	F	12	Unknown
13	M	11	Herniation of the sigmoid due to congenital mesenterial defect
14	F	24	Lung emboly
**40 weeks (*N* = 12)**			
15	M	40 weeks 2 days	Perinatal asphyxia
16	F	39 2/7 weeks 2 days	Perinatal asphyxia
17	M	40 weeks 1 day	Perinatal asphyxia
18	M	42 1/7 weeks 3 days	Perinatal asphyxia
19	F	41 weeks 10 days	Perinatal asphyxia
20	M	40 6/7 weeks 4 days	Perinatal asphyxia
21	M	40 2/7 weeks 1 days	Perinatal asphyxia
22	M	40 2/7 weeks 1 day	Perinatal asphyxia, congenital heart defect
23	M	40 2/7 weeks 4 days	Perinatal asphyxia
24	M	41 5/7 weeks 3 days	Perinatal asphyxia
25	F	35 weeks 1 day	Perinatal asphyxia, first born of dichorionic twin
26	M	41 6/7 weeks 7 days	Perinatal asphyxia

*Age is represented in years for the 25–70 and 10–25 years age groups and in weeks of gestation and postnatal days for the 40 weeks group (e.g., 41 6/7 w 7 = 41 weeks and 6 days of gestation whereafter the newly born lived for 7 days).*

### Sample Processing

Samples were obtained from the splenic hilar region and were cut in the transversal plane. Paraffin embedded splenic samples were cut on a microtome (Leica 2050 Super Cut, Nussloch, Germany) and 5 μm thick sections of splenic tissue were collected on glass slides, air dried and subsequently heat fixed for 2 h on a slide drying table of 60°C (Medax, 14801, Kiel, Germany). All slides were deparaffinized, rehydrated and further processed for histochemical or immunohistochemical staining. Hematoxylin/Eosin (HE) was used to evaluate technical tissue quality, to generate a tissue overview, and to screen for general pathological changes. A double T and B cell staining, using antibodies against specific membrane proteins, being CD3 and CD20 respectively, was used to screen the white pulp for distinct pathological abnormalities. To quantify and compare the overall presence of sympathetic nerves and T cells, and the distribution of sympathetic nerves in the PALS and other splenic structures (capsule, trabeculae, rep pulp and arteries), a double staining for sympathetic nerves and T cells was performed. In this procedure antibodies against CD3 and tyrosine hydroxylase (TH), were used, the latter being an enzyme involved in the synthesis of NE. The general nerve marker, protein gene product 9.5 (PGP9.5) was used on adjacent slides to confirm neural identity of TH-immune reactive (IR) structures.

### Staining Procedures

Tissue sections were dewaxed in xylene and rehydrated through graded alcohols prior to histochemical or immunohistochemical staining. Prior to immunohistochemistry, sections were pre-treated with Heat Induced Epitope Retrieval (HIER) in citrate buffer (pH6.0) for 20 min at 95°C.

#### Hematoxylin/Eosin Staining

Tissue sections were stained with hematoxylin for 10 min at room temperature (RT). After rinsing in running tap water, sections were dipped in ethanol 50%, stained with eosin for 1 min and dehydrated in graded alcohols and xylene. Slides were coverslipped with Entellan (Merck, Darmstadt, Germany).

#### Single Immunohistochemical Staining Procedures (PGP9.5 and Tyrosine Hydroxylase)

After the HIER procedure, sections were incubated with 5% Normal Human Serum (NHS) in TBS prior to incubation with rabbit anti human PGP9.5 antibody (1:2000 in TBS-T + 3% BSA, 48 h, 4°C, Dako, Glostrup, Denmark) or rabbit anti-human TH (1:1500 in TBT-T + 1% BSA, overnight RT, Pel-Freez, Rogers AR). Visualization of bound antibodies was performed with undiluted Brightvision Poly-Alkaline Phosphatase (AP) Goat-anti-Rabbit (ImmunoLogic, Amsterdam, Netherlands) and Liquid Permanent Red (LPR, Dako). All sections were counterstained with hematoxylin (Klinipath), dried on a hotplate for 15 min at 60°C and coverslipped with Entellan (Merck). Tris-buffered saline with 0.05% Tween20 (TBS-T) was used for all regular washing steps. Negative controls were obtained by incubation with TBS-3% BSA without primary antibodies. Human vagus nerve – and sympathetic trunk sections were included as a positive control for general – and sympathetic nerve tissue respectively. Both, staining procedures and positive controls, have been used in previous studies in which they proved to be valid to detect small nerves (including single nerve fibers) and to serve as proper controls, respectively (Cleypool et al., 2019, 2020).

#### Sequential Double Immunohistochemical Staining Procedure (CD20/CD3 and CD3/TH)

After HIER, sections were incubated with 3% Normal Goat Serum (NGS) (CD20/CD3) or 5% NHS (CD3/TH). In the first staining sequence, sections were incubated with CD20 or CD3 antibodies (details of used antibodies, including dilution, incubation time are presented in Table 2) and visualized with Brightvision Poly-AP Goat-anti-Mouse or Goat-anti-rabbit Mouse (ImmunoLogic) respectively followed by PermaBlue plus/AP (Diagnostics Biosystems, Pleasanton, United States). Details of the used antibodies, including dilution and incubation time are presented in Table 2. Prior to the second staining sequence a HIER in citrate buffer (pH6.0, 15 min RT) was performed, removing unbound antibodies but leaving chromogens unhanged (Van der Loos, 2010). Sections were then incubated with 3% NGS (CD20/CD3) or 5% NHS (CD3/TH) followed by incubation with CD3 and TH antibodies, where after they were visualized with Brightvision Poly-AP Goat-anti-Rabbit (ImmunoLogic) and LPR (Dako, Glostrup, Denmark). Sections were dried on a hotplate for 15 min at 60°C and coverslipped with Entellan (Merck, Darmstadt, Germany). Tris-buffered saline with 0.05% Tween20 (TBS-T) was used for all regular washing steps. Negative controls were obtained by incubation with TBS-3% BSA without primary antibodies. Human spleen sections that were previously confirmed to show proper staining for B cells, T cells and sympathetic nerves were included as positive controls.

**TABLE 2:** Detailed information on antibodies used in sequential double staining procedures.

**Double stain**	**Staining sequence**	**Primary antibody**	**Host**	**Vendor**	**Dilution, incubation time and temperature**	**Secondary antibody**	**Chromogen**
**CD3/TH**	1	CD3	Rabbit	Dako A0452	1:50, 90 min, RT	Brightvision-anti-Rabbit/AP	PermaBlue
	2	TH	Rabbit	Pel-Freez P40101	1:1500, overnight, RT	Brightvision-anti-Rabbit/AP	LPR
**CD20/CD3**	1	CD20	Mouse	Dako M0755	1:400, 90 min, RT	Brightvision-anti-Mouse/AP	PermaBlue
	2	CD3	Rabbit	Dako A0452	1:100, 90 min, RT	Brightvision-anti-Rabbit/AP	LPR

### Microscopic Evaluation

Hematoxylin/eosin and CD3 and CD20 stain was evaluated by bright field microscopy. The chromogen LPR was used to visualize sympathetic nerves. This marker has stable fluorescent characteristics and allows the user to alternately use bright field and fluorescent microscopy on the same slide. This can be beneficial as both modalities have their own advantages, e.g., fluorescent microscopy is more sensitive allowing small nerves to be more easily recognized, whereas bright field allowed better discrimination between lymphocytes and other cells. Instead of using a band pass filter suited for LPR, a long pass filter was used which allowed emission of a broader range of wave lengths. This resulted in a green/yellow autofluorescence of connective tissue, which was used to determine if the observed nerve extended beyond, e.g., perivascular connective tissue. All samples were studied using a DM6 microscope (Leica, Nussloch, Germany) with an I3 fluorescent filter.

### Image Acquisition

Single images were captured at various magnifications. These images were either brightfield or fluorescent images, depending on which modality appeared most suited to visualize the structures of interest. Both brightfield and fluorescent tile scans (stitched overlapping images) were captured for digital image analysis. Tile scans were either used to quantify the total amount of sympathetic nerves and T cells or to automatically select PALS regions which were then further studied in detail regarding their innervation (all tile scans were obtained using a 10x objective). Image acquisition was performed using a DM6 microscope with a motorized scanning stage, a I3 fluorescent filter, a DFC7000 T camera and LASX software (all from Leica, Nussloch, Germany).

### Quantitative Analysis of General Sympathetic Nerve and T Cell Presence

Bright field and fluorescent tile scans of CD3 and TH stained slides, respectively, were optimized and analyzed in Fiji (ImageJ with additional plugins) (Schindelin et al., 2012). Optimization of the images included removal of irrelevant tissue (e.g., hilar connective tissue and vasculature), artifacts and large trabecular arteries with large surrounding nerves. Both the total splenic tissue area and the area of TH and CD3-IR tissue were selected using standardized thresholds and data was expressed in pixels. The overall area occupied by sympathetic nerves and T cells was expressed as area% with respect to the total tissue area. Table 3 contains an overview of the different parameters investigated in this study, including a short description of the quantification method and how the data are expressed.

**TABLE 3:** Studied parameters, their method of quantification and data expression.

**Quantitative analysis of:**	**Method of quantification**	**Expressed as**
General sympathetic nerve presence	Automated counting of TH-IR pixels	Area %
General T cell presence	Automated counting of CD3-IR pixels	Area %

**Semi-quantitative analysis of:**	**Method of quantification**	**Expressed as**

# PALSs with sympathetic nerves	Automated selection of PALSs and manually counting of + PALSs	%
**Sympathetic nerve density in:**		
PALS	Microscopic evaluation of automated selected PALS for the relation of sympathetic nerves with T cells	Score 1–3
Capsule	General microscopic evaluation	Score 1–3
Trabeculae	General microscopic evaluation	Score 1–3
Red pulp	General microscopic evaluation	Score 1–3
Arteries	General microscopic evaluation	Score 1–3

### Semi Quantitative Analysis of Sympathetic Nerve Presence and Density in Various Splenic Areas

All samples contained large and small sympathetic nerves. Large nerves run with penetrating and trabecular arteries, whereas small nerves occur as discrete entities or are associated with smaller vascular structure from which they occasionally extent to the surrounding tissue. Only small nerves were evaluated in this study, since they are of relevance in regulation of local processes, such as immune cell function.

#### Periarteriolar Lymphatic Sheaths

For each sample a series of PALS regions was automatically selected and further studied in detail. No difference was made in types of PALS (being follicle associated PALS or non-follicle associated PALS). Automated selection was performed in Fiji, using tile scans of CD3/TH-stained slides. A threshold was set to select all CD3-IR areas, which were turned into solid regions using a blur function. Solid regions of 40.000 pixels or more were then selected. This approach resulted in a selection of 23–60 PALSs of substantial size. A maximum of 40 selected PALSs were then evaluated for the presence of sympathetic nerves. The number of positive PALSs was counted and expressed as percentage of the total number of studied PALSs. The association of sympathetic nerves with T cells was then graded as follows; 1: only one or two sympathetic nerves were observed to extend beyond the connective tissue of the vessel wall and to be in close proximity to T cells immediately lining the vessel wall, 2: multiple sympathetic nerves extended beyond the connective tissue of the vessel wall and were in close proximity to T cells immediately lining the vessel wall and 3: comparable to 2, but nerves extended beyond T cells immediately lining the vessel wall.

#### Capsule, Trabeculae, Red Pulp and Arteries

All samples were studied microscopically using a 20x objective and alternately switching between brightfield and fluorescent microscopy for reasons described in section “Microscopic Evaluation.” Nerve density in various splenic areas was quantified by means of scoring according to the following grading scale: 0: complete absence, 1: low density, 2: moderate density, and 3: high density. These scores were assigned when the observation was representative for the whole sample. With respect to the arteries, a division was made into large and small arteries. Large arteries represented penetrating arteries, also referred to as trabecular arteries (these were surrounded by a substantial amount of connective tissue). Small arteries represented arteries that could be observed in the red and white pulp.

Prior to scoring, various samples of the different age groups were evaluated by the observers in order to obtain a general idea of the extent of PALS innervation, the relation of PALS related sympathetic nerves with T cells, and to low and high nerve densities in other areas. Each sample was examined independently by two observers (CC and DB) who were blinded for the age group. When there was disagreement between the observers the samples were re-examined and scored by consensus.

### Statistical Analysis

Statistical analysis and graph conception were performed using Graphpad Prism 8. A Kruskall–Wallis test was used to compare the three age groups for their general sympathetic nerve and T cell presence, and, for sympathetic nerve density in various splenic areas. An uncorrected Dunn’s test was used to provide a *p*-value for each separate group comparison. All parameters were expressed as median followed by their inter quartile range. Association between the general presence of sympathetic nerves and T cells was tested by means of Pearson’s correlation coefficient.

## Results

**FIGURE 1:**
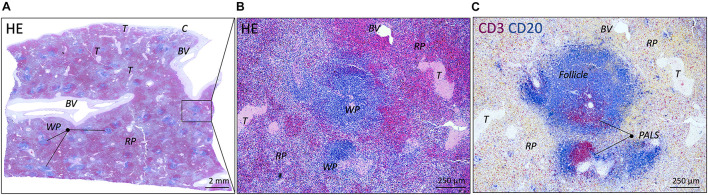
Microscopic images of a normal spleen. **(A)** Overview image of a splenic sample of a patient from the 10–20 years group (HE staining). White and red pulp can be clearly distinguished as well as vascular structures and connective tissue structures such as trabeculae. **(B)** Close up image of the boxed splenic region in **(A)**, showing normal splenic pulp morphology with clear white and red pulp areas (HE staining). **(C)** Similar region as in **(B)**, showing the presence of T and B cells (CD3 and CD20, respectively) in periarteriolar lymphatic sheaths (PALSs) and follicles, respectively. BV, blood vessel; C, capsule; WP, white pulp; RP, red pulp; T, trabecula.

All spleens showed well defined white pulp with distinct T and B cell regions (PALS and follicles, respectively) and red pulp with well-defined splenic cords red pulp sinusoids were more indistinctly present. All samples contained blood vessels of various sizes, trabeculae and most samples contained a significant bit of capsule. No pathological abnormalities were observed. TH-IR structures showed comparable patterns to PGP9.5-IR structures in adjacent slides, confirming their nerve identity. Figure 1 shows examples of normal splenic morphology.

### General Sympathetic Nerves and T Cells Presence and Their Age-Related Variation

**FIGURE 2:**
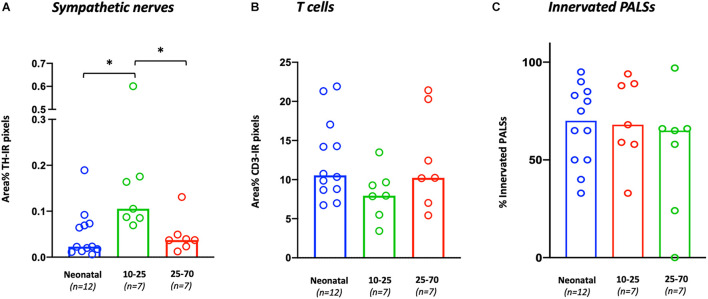
Age-related variations in general T cell and sympathetic nerve presence and the percentage of innervated PALS. **(A)** The general presence of sympathetic nerves per sample is calculated as the number of TH positive pixels and expressed as area % with respect to the total area of each sample. **(B)** The general presence of T cells per sample is calculated as the number of CD3 positive pixels and expressed as area % with respect to the total area of each sample. **(C)** The number of sympathetic innervated PALSs per sample expressed as percentage of the total selected number of PALSs in each sample. ^∗^*P* < 0.05.

Sympathetic nerves were detected in 26/26 samples (100%). Nerves were mostly observed surrounding vascular structures and to a lesser extent as discrete structures in the PALS, capsule, trabeculae and red pulp. T cells were observed in all samples and were primarily present in the PALS and to a lesser extent in follicles and in the red pulp. General sympathetic nerve presence was higher in the 10–25 group (0.1 [0.09–0.18]) compared to the neonatal group (0.02 [0.01–0.07], *p* = 0.0034) as well as compared to the 25–70 group (0.04 [0.02–0.05], *p* = 0.0192) (Figure 2A). No significant difference in T cell presence was observed between the different age groups (Figure 2B). No correlation was found between sympathetic nerve and T cell presence (*r* = 0.076, *p* = 0.71). Table 4 contains an overview of quantified median data per age group and lists age-related significant differences. Data of all separate individuals can be found in the Supplementary Data.

**TABLE 4:** Age related variations of T cell and sympathetic nerve presence.

	**Neonatal**	**10–25 years**	**25–70 years**	**Significant difference**
General sympathetic nerve presence (Area %)	*0.02*	*0.10*	0.04	10–25 >neonatal *p* = 0.0034
	*(0.01–0.07)*	*(0.09–0.18)*	*(0.02–0.05)*	10–25 >25–70 *p* = 0.0192
General T cell presence (Area %)	10.55	7.95	10.23	NS
	*(8.70–16.34)*	*(5.50–9.66)*	*(7.02–20.29)*	
# PALSs with sympathetic nerves (%)	70 *(50–95)*	68 *(58–89)*	65 *(24–66)*	NS
Score 1 (%)	38	34	21	Neonatal >25–70 *p* = 0.0059
	*(28.5–60)*	*(21–41)*	*(8–41)*	
Score 2 (%)	10	9	8	NS
	*(5–14.75)*	*(4–19)*	*(1–11)*	
Score 3 (%)	4	6	9	NS
	*(0–9.5)*	*(0–12)*	*(0–11)*	
**Sympathetic nerve density in:**				
Capsule	2	0	0	Neonatal >10–25 *p* = 0.0003
	(1–2)	(0–0)	(0–1.25)	Neonatal >25–70 *p* = 0.008
Trabeculae	1	1	1	NS
	*(1–2)*	*(1–3)*	*(1–2)*	
Large arteries	3	2	2	Neonatal >25–70 *p* = 0.0098
	*(2–3)*	*(2–3)*	*(1–2)*	
Small arteries	3	2	2	NS
	*(2–3)*	*(2–3)*	*(1–2)*	
Red pulp	2	1	0	Neonatal >25–70 *p* = 0.0016
	(1–2)	(0–2)	(0–1)	

*Data is expressed as median values (interquartile range is placed between brackets). Observed significant differences between groups are listed including their *p-*value.*

### Sympathetic Nerve Presence in the Periarteriolar Lymphatic Sheaths and Its Age-Related Variation

**FIGURE 3:**
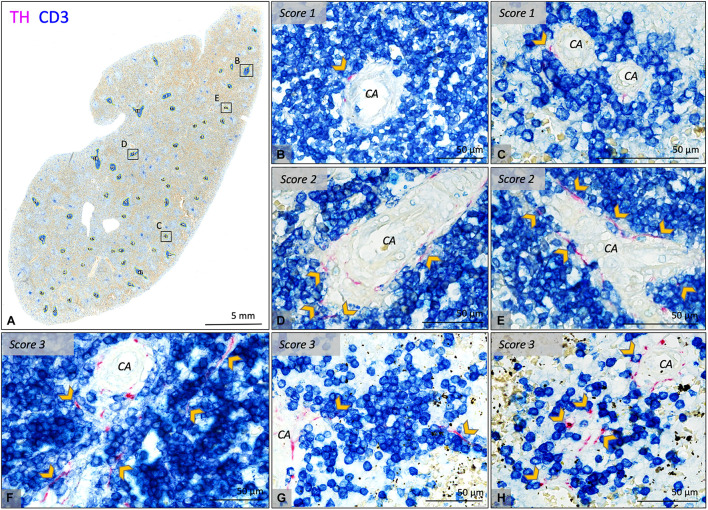
Bright field microscopic images of PALS related sympathetic nerves in CD3/TH double stained splenic tissue slides of various neonatal individuals. **(A)** Overview image of a splenic sample showing automatically selected T cell regions which were further investigated for the presence of sympathetic nerves and their relation with T cells. **(B–E)** Close up images of the marked regions in **(A)** representing PALSs with a score 1 or 2. **(F–H)** Close up images of PALSs with a score 3 (all from different neonatal individuals). Score 1: Only one or two sympathetic nerves were observed to extend beyond the connective tissue of the vessel wall of the central artery (CA) and to be in close proximity with T cells immediately lining the vessel wall. Score2: multiple sympathetic nerves extended beyond the connective tissue of the CA and were in close proximity with T cells immediately lining the vessel wall. Score 3: comparable to 2, but nerves extended beyond T cells immediately lining the vessel wall. CA, central artery; Arrow heads: pointing out sympathetic nerves that are in close proximity with T cells, but might be obscured by the blue stain.

In 25/26 subjects (96%), sympathetic nerves were occasionally observed to extend beyond the adventitial lining of the central artery into the lymphatic tissue where they were in close proximity to T cells. To gain an objectified understanding of the number of innervated PALSs, a series of PALSs was automatically selected for each sample and further studied in detail (Figure 3A). PALSs with sympathetic nerves in close proximity to T cells were observed in 614 of the in total studied 1027 PALSs (60%). No significant difference in the percentage of innervated PALSs was observed between the different age groups (Figure 2C). All PALSs that contained sympathetic nerves were additionally evaluated with respect to the number of nerves that were in association with T cells and whether these nerves would travel further into the lymphatic tissue. Of the in total 1027 studied PALSs, 302 (29%) PALSs contained one paravascular nerve in proximity to T cells (score 1), 249 (24%) PALSs contained multiple nerves (score 2) and 63 (6%) PALSs contained nerves which traveled further into the lymphatic tissue (score 3) (Figures 3B–H contains examples of all scores). The neonatal group showed a higher percentage of PALSs with a score 1 (38 [28.5–60]) compared to the 25–70 group (21 [8–41], *p* = 0.0059). For the other scores no age-related differences were observed.

### Sympathetic Nerve Density in Other Splenic Areas and Its Age-Related Variation

#### Capsule

**FIGURE 4:**
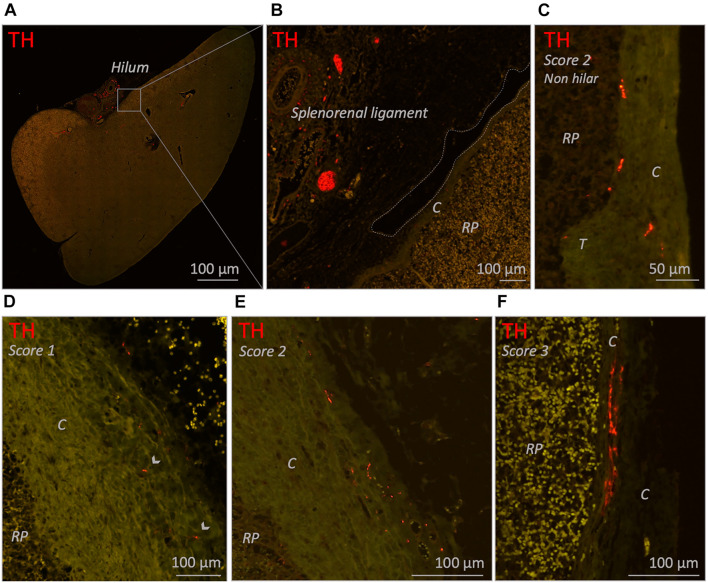
Fluorescence microscopic images of various capsule related sympathetic nerve densities (TH staining). **(A)** Overview image of a neonatal spleen. Large blood vessels with a perivascular nerve plexus reside in the splenorenal ligament and enter the spleen at the hilum. **(B)** Close up images of the boxed region in **(A)**. The splenorenal ligament contains connective tissue, vascular structures and sympathetic nerves. The lining of the splenorenal ligament reflects over the hilar capsule and on its distal continuation thins out (dotted line shows the lining of the ligament). **(C)** Close up image of a part of a capsule obtained from a non-hilar part of a neonatal spleen. A moderate density (score 2) of sympathetic nerves can be observed. **(D,E)** Close up images of hilar capsule samples of spleens of individuals from the 25–70 years age group containing a low (score 1) and moderate (score 2) density of sympathetic nerves, respectively. **(F)** Close up image of hilar capsule sample of a neonatal spleen with a high (score 3) density of sympathetic nerves. Capsular nerves were mostly observed in the more superficial and middle part of the capsule and only sporadically in the deeper part where they were in direct contact with the red pulp (as shown in **C**). C, capsule; RP, red pulp; T, trabecula. Arrow heads: small capsular nerves.

In 25/26 subjects (96%) a substantial amount of capsule was present of which 15 (60%) contained sympathetic nerves. These nerves were scattered and were mainly observed in the part of the capsule which was in close proximity to the hilum, where vascular structures with surrounding nerves entered the spleen. In the hilar region, the capsule was less distinct and showed continuity with hilar specific structures such as the adventitia of incoming vascular structures or the connective tissue of suspending splenic ligaments (Figures 4A,B). Most observed capsular nerve tissue was present in the more superficial and middle part of the capsule (Figures 4D–F) and only sporadically in the deeper part, where it was in direct contact with the red pulp (Figure 4C).

#### Trabeculae

**FIGURE 5:**
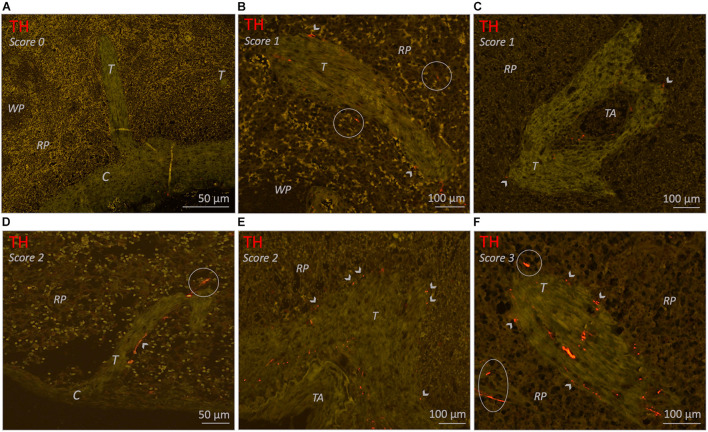
Fluorescence microscopic images of various trabecula related sympathetic nerve densities (TH staining) in different neonatal individuals. **(A)** Spleen without trabecular sympathetic nerves (score 0). **(B)** Trabecula with a low density (score 1) of sympathetic nerves. A few small nerves are present within the connective tissue of the trabecular and a few nerves can be observed on its outer margin where they are in proximity to the RP. **(C)** Trabecula with a low density (score 1) of sympathetic nerves. Perivascular nerves can be observed in the adventitia of a small blood vessel (trabecular artery) and in the connective tissue of the trabecula from where it diverges to its outer margins where a few nerves are bordering the RP. **(D)** Trabecula extending from the capsule with a moderate density (score 2) of sympathetic nerves. Most nerves are in proximity to the RP and on its cranial site a nerve extents into the RP. Sympathetic nerves in parts of trabeculae directly extending from the capsule were very sparse. **(E)** Comparable to **(C)** but this figure contains a larger trabecular artery and shows a moderate density (score 2) of sympathetic nerves. **(F)** Trabecula with a high density (score 3) of sympathetic nerves which diverge to the trabecula’s outer border to be in proximity to the RP.

All subjects showed trabeculae which, as a result of the cutting plane of the samples, were observed either as immediate extensions of the capsule, or as discrete structures deeper in the parenchyma (Figure 5A). The deeper parts of the trabeculae frequently contained large vascular structures (Figures 5C,E). In 25/26 (96%) subjects, trabecular sympathetic nerves were present to some extent and were often observed in the deeper parts of trabeculae, whereas the trabeculae that extended immediately from the capsule were mostly devoid of nerves. Trabecular sympathetic nerves were observed as discrete structures (Figures 5B,D,F), or as a nerve plexus surrounding vascular structures (Figures 5C,E). In both cases nerves could extend up to the external border of the trabecular tissue where nerves were in proximity to the surrounding red pulp.

#### Arteries

**FIGURE 6:**
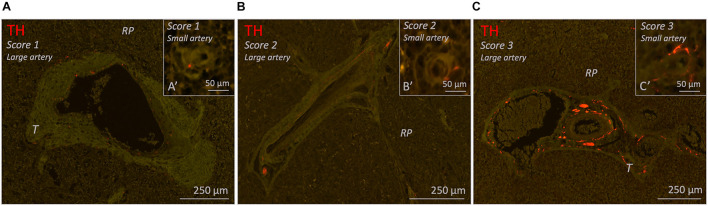
Fluorescence microscopic images of various of blood vessel related sympathetic nerve densities (TH staining) in different 25–70 years age group individuals. Each figure contains a representative example of a large vessel and a small vessel with a specific amount of sympathetic nerves (score 1–3). **(A–C)** Splenic samples with a low, moderate or high density of sympathetic nerves surrounding large arteries (score 1-3). (A’–C’) Splenic samples with a low, moderate, or high density of sympathetic nerves surrounding small arteries (score 1–3). RP, red pulp; T, trabecula.

All 26 subjects had clear recognizable vascular structures of various sizes which were to some extent surrounded with perivascular sympathetic nerves (Figure 6). In case of splenic artery branches in the hilum or large incoming trabecular arteries, nerve tissue was presented as large nerve bundles running in the adventitia or trabecular connective tissue, respectively, or as finer neural structures. In case of smaller arteries, nerves were organized in a more delicate network that were in close proximity to the vessel wall. Occasionally, perivascular nerves extended beyond the adventitial connective tissue. This was observed in central arteries, trabecular arteries and to a lesser extent in small arteries in the red pulp.

#### Red Pulp

**FIGURE 7:**
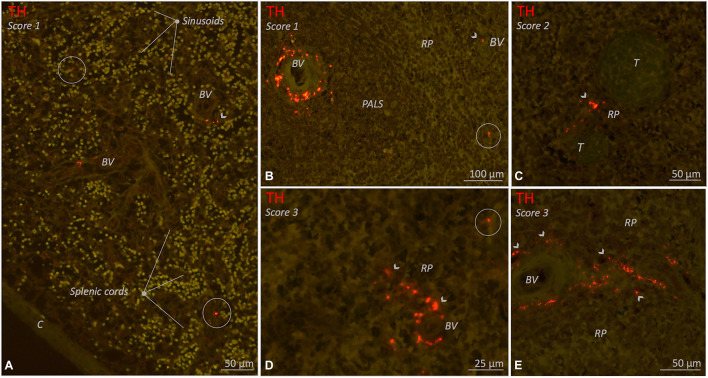
Fluorescence microscopic images of various red pulp related sympathetic nerve densities (TH staining) in different 10–25 year age group individuals. Sympathetic nerves are present as discrete structures running in the splenic cords (encircled structures) or as nerves that originate from a perivascular or trabecular plexus and from there diverge further into the red pulp (RP) (arrow heads). **(A)** Splenic sample with clear splenic cords and sinusoids and a low density (score 1) of sympathetic nerves. **(B–E)** Various examples of spleens with RP related nerves either as a low density (score 1), a moderate density (score 2) or a high (score 3) RP related sympathetic nerve density. C, capsule; BV, blood vessel; PALS, periarteriolar lymphatic sheath; RP, red pulp; T, trabecula.

The red pulp of all subjects contained clearly recognizable lymphoid tissue, also known as the splenic cords (Figure 7A). Sinusoids were easily recognized if they contained a substantial amount of blood, but otherwise were less distinct. Sympathetic nerves were observed in the red pulp of 20/26 (77%) subjects. These nerves comprised either small solitary nerves in between the red pulp (Figures 7B,D), or nerves bordering parenchymal trabeculae (Figures 5B,E,F, 7C) or small vascular structures (Figures 5F, 7A,D,E).

#### Age-Related Variations

Age-related differences were observed with respect to nerve density in the capsule, large arteries and the red pulp. The neonatal group showed a significant higher nerve density in its capsule compared to both the 10–25 and 25–70 group, and, surrounding its large arteries and in its red pulp when compared to the 25–70 group. No age-related differences were observed in trabeculae and small arteries. Table 4 contains detailed information on median group data and *p*-values.

## Discussion

This study shows that the human spleen contains sympathetic nerves, not only associated with the splenic vasculature, but also as discrete structures in the PALS, capsule, trabeculae and red pulp. Furthermore, the presence of sympathetic nerves shows a mild tendency to decrease with age. These findings are of relevance for understanding the role of splenic sympathetic nerves in regulation of the systemic immune response in humans and for the development of neuromodulatory anti-inflammatory therapies.

Sympathetic nerves were observed in all spleens but their presence was most prominent in the 10–25 age group, suggesting that from birth their number increases whereafter it decreases from adulthood on. This observation fits in with the fact that organ systems, including the peripheral nervous system, mature after birth until the onset of adulthood whereafter they subsequently show signs of aging (Verdú et al., 2000). More specifically, animal studies have shown that an age-related decline applies for splenic sympathetic innervation as well (Bellinger et al., 1987, 1992; Madden et al., 1997).

A decrease in T cell presence has been put forward as another explanation for a decrease in sympathetic nerve abundance (Hoover et al., 2017). The authors observed the presence of T cells to correlate to that of sympathetic nerves and suggested the lack of specific nerve growth factors produced by these T cells to be of relevance. In the current study, however, no correlation between the general presence of sympathetic nerves and T cells (both expressed as area%) was found, thereby further emphasizing aging to be the most plausible explanation for the observed decline of sympathetic innervation in spleens of healthy persons.

In 96% of the studied individuals, PALSs were observed to contain sympathetic nerves that extended beyond the adventitia of central arteries and to be in apposition with T cells. So far PALS innervation in humans have only been reported once (Hoover et al., 2017). The authors, however, did not supply information on the number of innervated PALSs per individual, deeming it impossible to estimate whether PALS innervation represented a structural entity of normal healthy spleens or a more coincidental heterogenous finding. The current study shows that human PALSs innervation was observed in 60% of the in total 1027 studied PALSs and therefore represents a structural phenomenon. Furthermore, no significant age-related differences could be determined with respect to the innervation of the number of PALSs, or the extent to which sympathetic nerves were in proximity to T cells.

Sympathetic innervation patterns observed so far in human PALSs, however, seem to differ significantly from rodent species. In rats, mice and rabbits, nerves were more densely present and also traveled further into the parenchyma (Felten et al., 1987, 1997; Bellinger et al., 1992). This questions whether splenic plexus stimulation in humans, with only a few T cells of the PALS in direct contact with sympathetic nerves, would target enough of these cells to establish a similar systemic anti-inflammatory effect as observed in rodents. It is, however, known that vagus nerve stimulation in humans results in a systemic anti-inflammatory response (Koopman et al., 2016). Since vagus nerve stimulation activates the splenic plexus (Reardon, 2016; Pavlov and Tracey, 2017) this effect must be elicited by splenic sympathetic nerves, potentially involving different components and/or locations than what is known for the prevailing CAIP NE-Ach-TNF mechanism. In the following part various alternative options for explaining this effect will be discussed in the light of our findings.

Most T cells are migratory cells and reside in the PALS for only a certain amount of time whereafter they disseminate to the red pulp and return to the systemic circulation. If, during this migration, enough CD4^+^T cells pass sympathetic nerves and short term synaptic connections are formed, a phenomenon referred to as short term plasticity (Song et al., 2018), a significant amount of adrenergic receptors on CD4 ^+^T cells might indeed get activated. Another potential mechanism to overcome this issue might be volume transmission; a process wherein a neurotransmitter is not released in a synaptic cleft but is expelled into the extracellular matrix and reaches its effector cells by diffusion (Fuxe et al., 2013). Volume transmission of NE could result in adrenergic activation in increased numbers and more distant T cells other than the ones in direct contact with sympathetic nerves. Furthermore, the splenic white pulp appears to contain low amounts of acetylcholinesterase (Hoover et al., 2020), and volume transmission of Ach (secreted by activated T cells in the PALS) might occur as well, thereby further expanding the indirect effector scope of sympathetic nerves.

Other studies have casted doubt on the prevailing mechanisms of the cholinergic anti-inflammatory pathway involving the sequence of NE release, ACh production, and subsequent TNFα reduction through cholinergic receptor activation on macrophages, both with respect to the mechanism itself and with respect to its location (Rosas-Ballina et al., 2008; Murray et al., 2017). In a recent study wherein the relationship between sympathetic neurons and ChAT^+^ lymphocytes were mapped in complete mouse spleens, it was shown that overall few ChAT^+^ T cells were juxtaposed to sympathetic fibers and that their distance to these fibers exceeded that of traditional synapses (Murray et al., 2017). Moreover, the authors showed that sympathetic innervation was involved in homing of ChAT^+^T cells to appropriate physical regions in the spleen by increasing the expression of the chemokine CXCL13 in stromal cells (Murray et al., 2017). Such homing processes are vital as it conjoins the right cells for a properly aligned immune response (reviewed by Zhao et al., 2015). In case of conjoining the key mediators of the intrasplenic NE-ACh-TNFα mechanism, this requires homing of ChAT^+^T cells towards macrophage rich areas such as the marginal zone and the red pulp, where adrenergic receptors on these T cells need to be activated prior to release of ACh in proximity to these macrophages. Interestingly, vagus nerve stimulation in mice specifically attenuated TNFα production by splenic macrophages in these two areas 30 minutes after endotoxin administration (Rosas-Ballina et al., 2008). The authors observed nerve terminals adjacent to these TNFα producing macrophages, but did not provide information on the local presence of ChAT^+^ T cells. With the above discussed topics in mind, it would be more plausible that the intrasplenic NE-Ach-TNFα mechanism occurs in the marginal zone and red pulp, instead of the PALS. Further support for favoring the red pulp and marginal zone over the PALS as designated immune regulation areas, is the difference in T cell transit time. T cell passage through the red pulp and marginal zone takes 5 and 50 min respectively whereas passage through the PALS (from the perivascular area to the macrophage rich marginal zone/red pulp) takes 2.5–6 h (Hammond, 1975; Ford, 1979; Ganusov and Auerbach, 2014) and the latter may take too long to provoke the fast systemic response which peaks at 90 min after electrical stimulation (Rosas-Ballina et al., 2008; Komega et al., 2018; Guyot et al., 2019).

According to recent literature, human spleens do not have a marginal zone but their red pulp is considered to be morphologically and functionally comparable to mice and rats (reviewed by Steiniger, 2015). In contrast to previous studies (Heusermann and Stutte, 1977; Kudoh et al., 1979; Anagnostou et al., 2007; Verlinden et al., 2018) the current study showed that, although rare, red pulp innervation is present in all age groups albeit it more prominent in younger individuals. Sporadically, discrete nerves were observed within the red pulp, but most of the red pulp innervation was supplied either by trabecular nerves which extended to the outer margins of the trabecular connective tissue, or by nerves positioned outside the connective tissue surrounding small red pulp vascular structures. These nerves always remained in proximity to the trabeculae and vascular structures and never traveled deeper into the red pulp. In human fetuses, capsular nerves have also been observed to extend into the red pulp (Anagnostou et al., 2007). In the current study, however, capsular nerves have been observed in all age groups, but only rarely extended into the red pulp or reached the inner capsular margins contacting the red pulp and therefore were not considered to generally contribute to red pulp innervation.

Thus, comparable to the PALS, the red pulp in humans contains significant less sympathetic innervation when compared to other animals in which red pulp innervation was already considered sparse (Bellinger et al., 1987). Therefore, again one could question whether splenic plexus stimulation in humans would target a sufficient amount of red pulp effector cells to provoke the effect observed in animals (Rosas-Ballina et al., 2008). While searching the literature for the role of the stromal cells in immune regulation, a more elegant and subtle mechanism, which potentially requires little direct innervation of red pulp immune cells, was found. In the spleen stromal cells reside in both the white and red pulp where they represent the main cellular components of the reticular framework, a connective tissue scaffold which provides support for splenic immune cells and guidance for their migration (Perez-Shibayama et al., 2019). As shown by a transmission electron microscopic study in guinea pigs, the reticular framework is composed enveloping reticular cells which enclose connective tissue components and occasionally a sympathetic axon or free nerve endings (Saito, 1990). The connective tissue space of the framework was shown to be continuous and to contain meshwork like spaces (Saito, 1990). The author referred to these meshwork like spaces as catecholamine canals since he hypothesized them to facilitate diffusion of released sympathetic neurotransmitters over larger distances throughout the reticular framework. With the exception of follicles, the reticular cells of the reticular framework are represented by contractile myofibroblasts (Pinkus et al., 1986). Contraction of these myofibroblasts results in exposure of migrating immune cells to the content of catecholamine canals; sympathetic nerve endings or previously secreted and diffused NE (Saito, 1990). Adrenergic signaling can than modulate the immune response, which in case of diffused NE does not require direct immune cell innervation. The reticular framework might equal the more recent discovered splenic conduit system in mice; an interconnected tubular network that functions as a transport system for fluid, small molecules and particles (including antigens) and is covered with fibroblast reticular cells which support migratory lymphocytes (Nolte et al., 2003; Roozendaal et al., 2008). Whether the human spleen contains catecholamine cannals or a conduit system has not been established yet.

Overall, it can be concluded that, apparent age-related and interspecies differences in splenic sympathetic nerve distribution and density exists. It is, however, uncertain if and to what extent these differences are of significance for NE-ACh-TNFα mechanism based anti-inflammatory therapies in humans. Although experimental studies have shown an indisputable role for sympathetic nerves, ChAT^+^ T cells and macrophages, it is, however, not completely understood how and where the various elements of the prevailing intrasplenic NE-ACh-TNFα mechanism interact and whether unknown intermediate elements are required. In order to be able to extrapolate experimental data to humans and to estimate whether targeting splenic sympathetic nerves in humans could be beneficial, additional experimental and morphological studies are required and alternative or additional mechanisms should be taken into consideration.

### Study Limitations

The use of single tissue sections will result in a 2D representation of sympathetic nerves in relation with surrounding structures. This makes it difficult to truly estimate whether the evaluated sympathetic nerves represent small, local tissue innervating (discrete) nerves or that they might be part of larger *en route* nerves. This bias should be kept in mind when interpretating data on sympathetic nerve quantification. However, since the same bias applies to all samples, we consider its influence on group comparison data negligible.

## Conclusion

Although less extensive when compared to other animals, human spleens contain sympathetic nerves, not only associated with vascular structures but also as discrete entities. In the PALS, these nerves were in proximity to T cells, suggesting the potential existence of a CAIP in humans. Alternative locations involved in neuroimmune regulation might be represented by the capsule, trabeculae and red pulp since these structures contain discrete sympathetic nerves as well. Since splenic sympathetic nerve distribution and density shows interspecies variation and our general understanding of the relative and spatial contribution of splenic innervation in immune regulation is incomplete, it remains difficult to estimate the anti-inflammatory potential of targeting splenic sympathetic nerves in humans. Future studies should focus on the anti-inflammatory efficacy of targeting these nerves in humans and further characterize the underlying mechanism. Splenic sympathetic innervation density slightly decreases from adulthood on and these age-related variations might be of relevance when developing sympathetic nerve based anti-inflammatory therapies.

## Data Availability Statement

The original contributions presented in the study are included in the article/Supplementary Material, further inquiries can be directed to the corresponding author/s.

## Ethics Statement

The studies involving human participants were reviewed and approved by the Medical Ethical Committee (#18-167) as a “non-Medical Research Act” study and the Biobank of the University Medical Center Utrecht approved to use the rest biomaterial for this research (biobank #18-284). Written informed consent to participate in this study was provided by the participants’ legal guardian/next of kin.

## Author Contributions

CC: study design, sample preparation, image analysis, data interpretation, and manuscript writing. DB: study design, sample preparation, data interpretation, and manuscript writing. MN, WJ, and RB: manuscript revision. PN, ML, and RB: sample collection and manuscript revision. RB: manuscript revision and project supervision. All authors contributed to the article and approved the submitted version.

## Conflict of Interest

The authors declare that the research was conducted in the absence of any commercial or financial relationships that could be construed as a potential conflict of interest.

## Publisher’s Note

All claims expressed in this article are solely those of the authors and do not necessarily represent those of their affiliated organizations, or those of the publisher, the editors and the reviewers. Any product that may be evaluated in this article, or claim that may be made by its manufacturer, is not guaranteed or endorsed by the publisher.

## Supplementary Material

Supplementary Table 1 Data of separate individuals.

## Abbreviations

ACh: acetylcholine
AR: adrenergic receptor
β: beta
BV: blood vessel
C: capsule
CAIP: cholinergic anti-inflammatory pathway
ChAT: choline acetyl transferase
HE: hematoxylin/eosin
HIER: heat induced epitope retrieval
IR: immune reactivity
NE: norepinephrine
PALS: periarteriolar lymphatic sheath
PGP9.5: protein gene product 9.5
RP: red pulp
T: trabecula
TH: tyrosine hydroxylase
TNF α: tumor necrosis factor alpha.
